# Medical Reform

**Published:** 1834-04-01

**Authors:** 


### MEDICAL POLITICS AND INTELLIGENCE.

### MEDICAL REFORM.

THE subject of Medical Reform is one of such vast extent, as well as of such deep importance, that we confess we approach it with the greatest diffidence. To draw up a scheme for re-casting the practice of physic throughout the British empire would require so profound a knowledge, not merely of professional minutiæ, but of human nature itself, that we shrink from the task; and, instead therefore of proposing some plan for a medical millennium fire-new from the mint of our imagination, we shall content ourselves with mentioning a few among the defects of the present system, together with such remedies as reason, the instincts of society, and justice—good-humoured justice, seem to demand.

The first on the list of alleged grievances is the supposed monopoly of the College of Physicians; but we should be glad to know in what this monopoly consists? It is true that the governors of hospitals and dispensaries in London have thought fit to exclude physicians who are not members of the College from holding office in those institutions. But surely in this case the monopoly, if it can be called one, is of the most laudable kind, for it is conferred not by force, but by merit. In this immense city, to which suspicious characters of all kinds flock by a sort of natural instinct, it seems reasonable to prefer the stamp of genuineness implied in being a member of the first medical society in the world, to the simple fact of being in possession of a diploma from some unheard-of university. No one can say that on this point the College has displayed any severity, or any desire to make the list of members of a monopolizing brevity. On the contrary, the fastidious would be inclined to reproach it with too good-humoured a facility—with admitting (as we find it stated in a late semi-official document,) the possessor of a degree from any university on earth: the alumnus of Aberdeen (whose diploma in one sense is worth its weight in gold,) is not rejected, and yet the spirit of monopoly is imputed to the College! Lord Durham indeed, in a speech, at the bottom of which he ought to have put *Medical Gazette*, as we do in our "Collectanea," endeavoured to ground the accusation of monopoly on the fact, that the proportion of physicians to the population of London is much smaller than in the cities of the continent: not knowing, probably, that London has at least three times as many physicians as it employs.

This naturally leads us to another topic. The majority of the sick in this country are attended by those who are at once physicians and druggists, who practise physic as a trade, and who, whatever may be their merits or their utility, can scarcely be expected to advance the art which they profess. What is the reason of this, and what is the remedy? Both are as clear as daylight, though not hinted at in Lord Durham's speech. The middle classes can pay but middling prices; and, if we wish Oxford street and Tottenham-Court road to have the benefit of medical advice, without swallowing barrels of pills, and hogsheads of saline draughts, we must encourage a race of physicians whose modest hopes will be commensurate with the less exuberant wealth of those who are to fulfil them; or, in plain English, we must have men who will take plebeian silver instead of patrician gold, and pocket a crown-piece without blushing.

Among the evils engendered by the junction of pharmacy and physic, there is one with which the most uninstructed part of the public are familiar—we mean the superfluity of medicine (and very nasty medicine too,) which is sent, swallowed (?), and paid for (? ?). "Those medicines," says Gray, "must, in most cases, be made unpalatable, lest the patient should conceive himself to be furnished with mere slops for the sake of a charge being made." (Supplement to the Pharmacopœia; Preface, p. x.) There is another monstrosity, however, derived from the same source,—from the conversion of physic into a trade, with which the public are less acquainted, namely, the selling of a practice. This driving of the sheepish patients, with sundry restive exceptions, from the pen of Mr. Savine into the pen of Mr. Calomelet, is really very ludicrous, and exhibits the infinite advantages of what Chesterfield calls a modest assurance. Mr. Savine, for 2000*l*. paid, or promised, endows his young successor with every medical virtue under heaven: the sick swallow the gilded bait, and form the live stock of the happy purchaser. Our pages are probably but rarely perused by unprofessional persons; but some of those publications which are the miracle of the age, and count their readers by myriads, should inform all whom it may concern, that the seller's loud praises of the purchaser mean nothing—that they are like an advocate's profession of his belief in the justice of his cause; or, to use a more poetical comparison, that they are like "the perjuries of lovers, at which Jove laughs, and orders the winds to bear them along, and dissolve them in their course."*

The apothecary's art, again, is an evil; and the manner in which it has been enforced of late is highly discreditable to the Company. This is one of the few cases in which the legislature can be of service to us: a simple repeal of the Act would relieve the profession from the disgraceful anomaly of being governed by a knot of traders, and leave it, as it ought to be, free as air.

This is the most essential point of all; and with this we conclude. Should the medical reformers of the day succeed in procuring an Act "for the more effectual suppression of unlicensed practitioners," let them rest assured that such an Act must, and ought, to be ineffectual. The preventive police, by which irregular practitioners may be put down under despotic governments, would not be tolerated in England; and, to punish infringements of such an act by means of informers, could be done only by violating all the charities, and even all the decencies of life, and would be worse than the death of a hundred patients by scarifying liniments or Hygienic pills.

---

* —— Perjuria ridet amantûm
Jupiter, et ventis virita ferre jubet. Ovid.

